# Incidence and OR team awareness of “near-miss” and retained surgical sharps: a national survey on United States operating rooms

**DOI:** 10.1186/s13037-021-00287-5

**Published:** 2021-04-03

**Authors:** Samuel A. Weprin, Dielle Meyer, Rui Li, Umberto Carbonara, Fabio Crocerossa, Fernando J. Kim, Riccardo Autorino, John E. Speich, Adam P. Klausner

**Affiliations:** 1 Division of Urology, Department of Surgery, VCU Health, Richmond, VA USA; 2 Division of Urology Denver Health Medical Center and University of Colorado Anschutz Medical Campus, Denver, USA

**Keywords:** “Near-miss”, Retained surgical item, Foreign body, Lost needle, Surgical sharps

## Abstract

**Introduction:**

A retained surgical sharp (RSS) is a never event and defined as a lost sharp (needle, blade, instrument, guidewire, metal fragment) that is not recovered prior to the patient leaving the operating room. A “near-miss” sharp (NMS) is an intraoperative event where there is a lost surgical sharp that is recovered prior to the patient leaving the operating room. With underreporting of such incidents, it is unrealistic to expect aggressive development of new prevention and detection strategies. Moreover, awareness about the issue of “near-miss” or retained surgical sharps remains limited. The aim of this large-scale national survey-based study was to estimate the incidence of these events and to identify the challenges surrounding the use of surgical sharps in daily practice.

**Methods:**

We hypothesized that there was a larger number of RSS and NMS events than what was being reported. We survived the different OR team members to determine if there would be discordance in reported incidence between groups and to also evaluate for user bias. An electronic survey was distributed to OR staff between December 2019 and April 2020. Respondents included those practicing within the United States from both private and academic institutions. Participants were initially obtained by designating three points of contact who identified participants at their respective academic institutions and while attending specialty specific medical conferences.

Together, these efforts totaled 197 responses. To increase the number of respondents, additional emails were sent to online member registries. Approximately 2650 emails were sent resulting in an additional 250 responses (9.4% response rate). No follow up reminders were sent. In total, there were 447 survey responses, in which 411 were used for further analysis. Thirty-six responses were removed due to incomplete respondent data. Those who did not meet the definition of one of the three categories of respondents were also excluded. The 411 were then categorized by group to include 94 (22.9%) from anesthesiologist, 132 (32.1%) from resident/fellow/attending surgeon and 185 (45%) from surgical nurse and technologist.

**Survey:**

The survey was anonymous. Participants were asked to answer three demographic questions as well as eight questions related to their personal perception of NMS and RSS (Fig. 1). Demographic questions were asked with care to ensure no identifiable information was obtained and therefore unable to be traced back to a specific respondent or institution. Perception questions 4–6 and 11 were designed to understand the incidence of various sharp events (e.g. lost, retained, miscounted). Questions 7 and 10 were dedicated to understanding time spent managing sharps and questions 8 and 9 were dedicated to understanding the use x-ray and its effectiveness.

**Results:**

Overall, most of each respondent group reported 1–5 lost sharp events over the last year. Roughly 20% of surgeons believed they never had a miscounted sharp over the last year, where only 5.3% of anesthesiologist reported the same (*p* = 0.002). Each group agreed that roughly 4 lost events occur every 1000 surgeries, but a significant difference was found between the three groups regarding the number of lost sharps not recovered per 10,000 surgeries with anesthesiologist, surgeon and nurse/technologist groups estimating 2.37, 2.56 and 2.94 respectively (*p* = 0.001). All groups noted x-ray to offer poor effectiveness at 26–50% with 31-40 min added for each time x-ray was used. More than half (56.8%) of surgeons reported using x-ray 100% of the time when managing a lost sharp whereas anesthesiologists and nurses/technologists believe it is closer to 1/3 of the time. An average of 21-30 min is spent managing each NMS, making a lost sharp event result in up to 70 min of added OR time.

**Conclusions:**

**“**Near-miss” and RSS are more prevalent than what is reported in current literature. Surgeons perceive a higher rate of success in retrieving the RSS when compared to anesthesiologists and OR nurses/technologists. We recognize several challenges surrounding “near-miss” and never events as contributing factors to their underreported nature and the higher degree of surgeon recall bias associated with these events. Additionally, we highlight that current methods for prevention are costly in time and resources without improvement in patient safety. As NMS and RSS have significant health system implications, a strong understanding of these implications is important as we strive to improve patient safety.

## Background

The idea of safe practice in Patient Safety Culture (PSC) has been an important aspect for advancement in health care since its introduction in the Institute of Medicine report “To Err is Human” [1]. PSC embodies properties in patient care guidelines and processes that are crucial for preventing adverse events in health care. Organizational properties for PSC extend beyond the number of incidents in patient care processing and include established safety values and knowledge of specific patient care protocols [2].

Although the practice of PSC has become popular, retained surgical items (RSI) still represent the most frequently encountered sentinel event in operating rooms worldwide [3]. However, there is still a paucity of literature describing the incidence of what are defined as “*near-miss”* and *retained* surgical sharps [4].

A *retained* surgical sharp (RSS) is a never event and is defined as a lost sharp (needle, blade, instrument, guidewire, metal fragment) that is not recovered prior to the patient leaving the operating room. The term “Never Event”, as defined by the National Quality Forum, is a medical error that should never occur, usually preventable and results in significant patient harm (e.g. wrong-site surgery) [x]. These must be reported to the patient as well as to the hospital incident committee. In certain cases, this is discovered in the post anesthesia recovery unit (PACU) during a routine x-ray. In other cases, the RSS is uncovered when the patient has an acute or chronic complication from the retained foreign body. There are also several cases of RSS being found incidentally, sometimes years later, during routine imaging for an unrelated condition [5–8].

A “*near-miss”* sharp (NMS) is an intraoperative event where there is a lost surgical sharp that is recovered prior to the patient leaving the operating room. A common example of a NMS is when a needle is lost inside the patient or in transition between the surgeon and the surgical assistant. In some cases, this is not recognized until the surgical count is incorrect at the end of the procedure, prompting the difficult question of whether the lost sharp could be retained inside the patient. Surgical teams will go to great lengths to recover any lost sharp given the increased risk to the patient, which can be a time-consuming and costly event.

Both NMS and RSS events are difficult to quantify and their reporting to the Joint Commission (JC) is voluntary, so that underreporting remains a reasonable concern. Moreover, NMS are even less likely to be voluntarily reported as the problem was rectified prior to the patient leaving the OR and therefore the degree of risk to the patient (and provider/hospital) has been significantly reduced. Furthermore, there is no easy or efficient way to report “near-miss”/never events directly to the JC and any reporting to one’s own administration typically results in significant paperwork, legal counsel and quality control measures - further reducing the likelihood of self-reported events. With underreporting of such incidents, it is unrealistic to expect aggressive development of new prevention and detection strategies. Moreover, awareness about the issue of “near-miss” or retained surgical sharps remains limited.

The aim of this large-scale national survey-based study was to estimate the incidence of these events and to identify the challenges surrounding the use of surgical sharps in daily surgical practice.

## Materials and methods

### Study design and population

Our IRB approved survey was distributed electronically to OR staff between December 2019 and April 2020. Respondents included those practicing within the United States from both private and academic institutions. Participants were initially obtained by designating three points of contact who identified participants at their respective academic institutions and while attending specialty specific medical conferences. Together, these efforts totaled 197 responses. To increase the number of respondents, additional emails were sent to online member registries. Approximately 2650 emails were sent resulting in an additional 250 responses (9.4% response rate).

In total, there were 447 survey responses, in which 411 were used for further analysis. 36 responses were removed due to incomplete respondent data. The 411 were then categorized by group to include 94 (22.9%) from anesthesiologist, 132 (32.1%) from resident/fellow/attending surgeon and 185 (45%) from surgical nurse and technologist.

### Survey

**Fig. 1:**
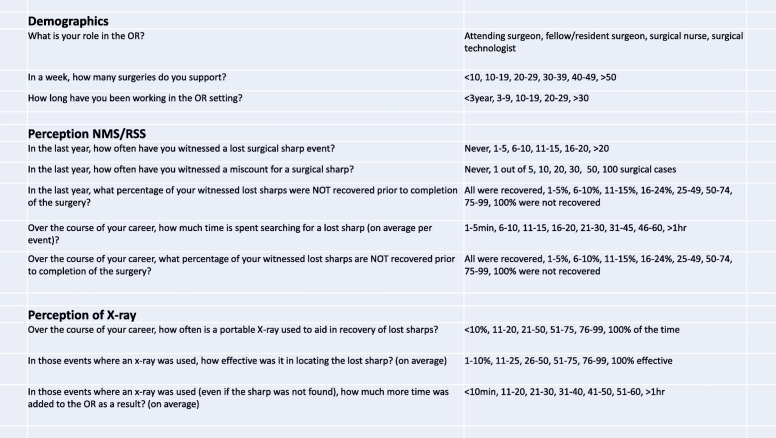
Survey Questions

The survey was anonymous. Participants were asked to answer three demographic questions as well as eight questions related to their personal perception of NMS and RSS (Fig. 1). Demographic questions were asked with care to ensure no identifiable information was obtained and therefore unable to be traced back to a specific respondent or institution. Perception questions 4–6 and 11 were designed to understand the incidence of various sharp events (e.g. lost, retained, miscounted). Questions 7 and 10 were dedicated to understanding time spent managing sharps and questions 8 and 9 were dedicated to understanding the use x-ray and its effectiveness.

### Analysis

Survey responses were ranked accordingly and statistically analyzed as appropriate in SPSS version 26. Kruskal-Wallis 1-way ANOVA test was employed to test the agreement of responses in three groups and pairwise comparisons were performed using Dunn’s procedure with a Bonferroni correction for the response, then Fisher’s exact test was used to examine the statistical difference on a portion of the specified responses in a pairwise comparison. Linear regression was used to derive the odds ratio. Incidence of NMS and RSS per surgical volume was estimated by using the median value of each response divided by the median number of surgeries in the respondent’s group. Results were expressed as mean scores.

To further our understanding of the discordance between groups, surgeons were compared to a combined group of anesthesiologist, nurses and technologist, and a binomial logistic regression was performed to assess the likelihood that the following events were reported by surgeons:

1. No surgical sharps were lost in the last year
2. A miscount of sharps happened in > 2% of surgeries in last year
3. 100% of lost sharps were recovered prior to completion of the surgery in the last year
4. A portable X-ray was used 100% of the time to aid in recovery of lost sharps

## Results

### Baseline demographics

Respondent demographics are presented in Table 1. By role, the median ranked value for the number of surgeries performed each week was 10–19 by surgeons and 20–29 by anesthesiologists and nurses/technologists. The median score for the number of years working in the OR was consistent among all three groups as 10–19 years.

**Table 1:**
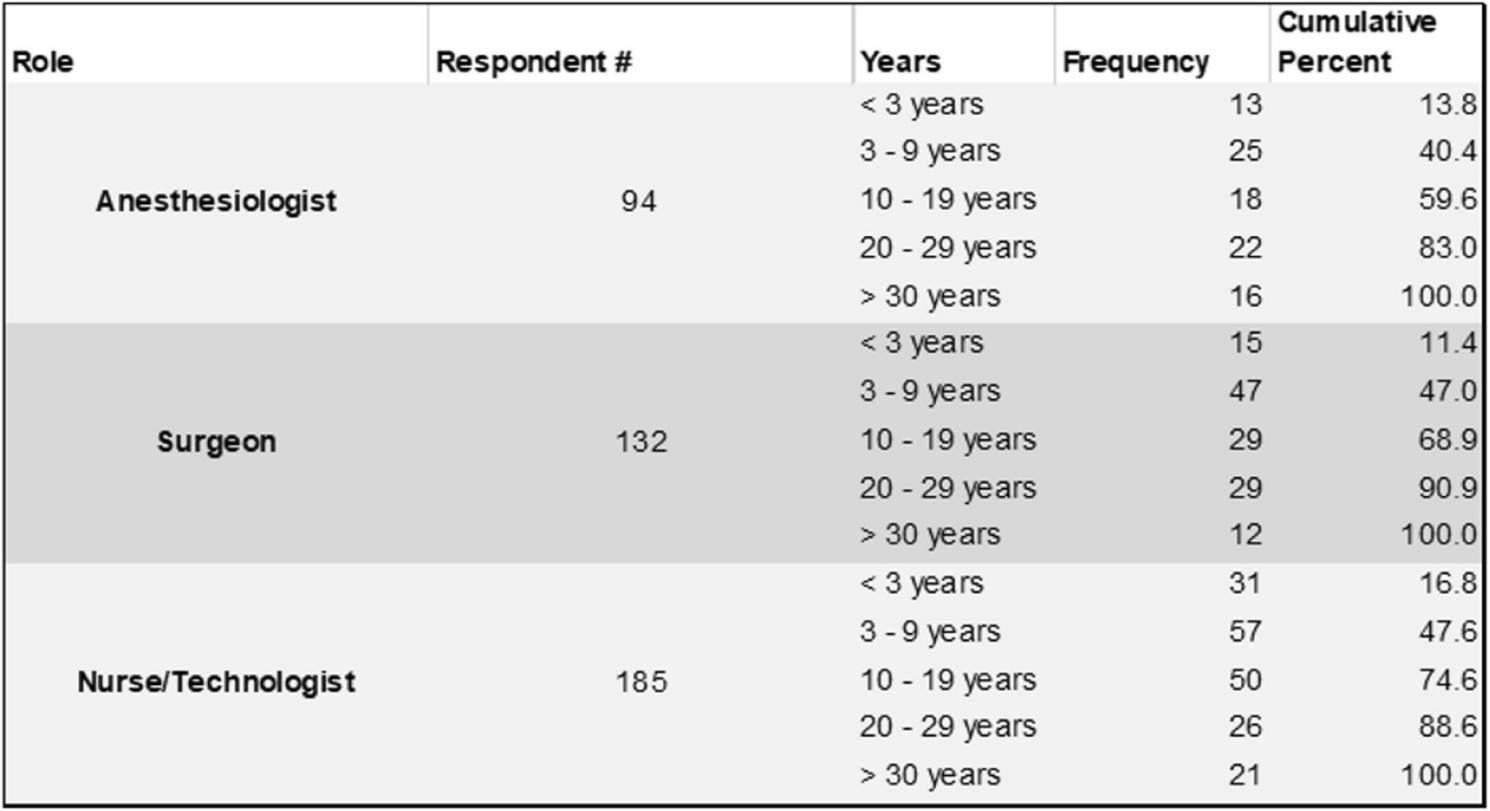
Demographics

### Overall perception of NMS and RSS

The distribution of ranked response scores was significantly different between the three groups in question 4, 5, 6, 8, 11 but similar in questions 7, 9 and 10. Pairwise comparison revealed statistically significant differences in median scores between the surgeon and anesthesiologist groups, and surgeon and nurse/technologist groups in most of questions, but not between the anesthesiologist and nurse/technologist groups (Table 2).

**Table 2:**
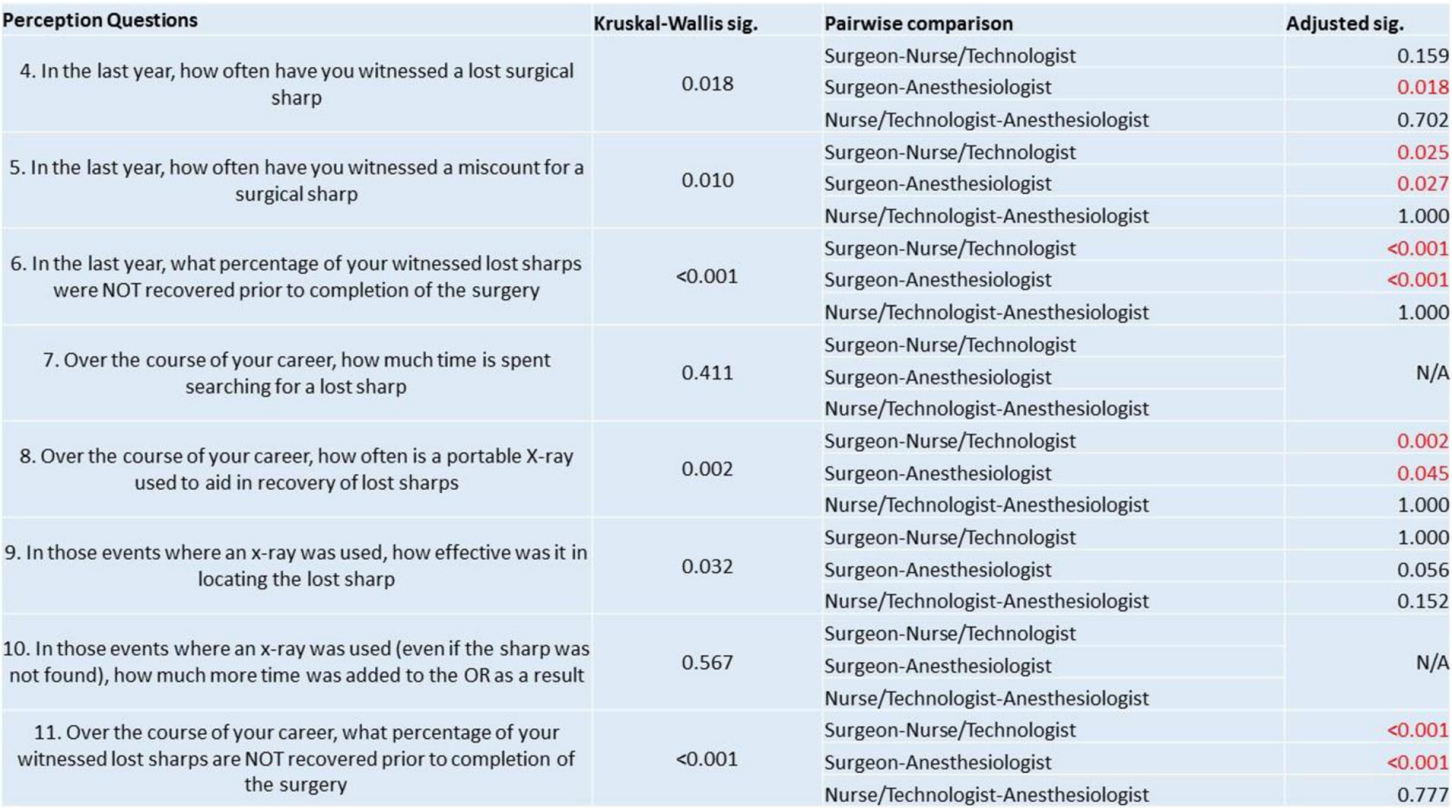
Distribution of ranked responses

### Incidence of events

**Fig. 2:**
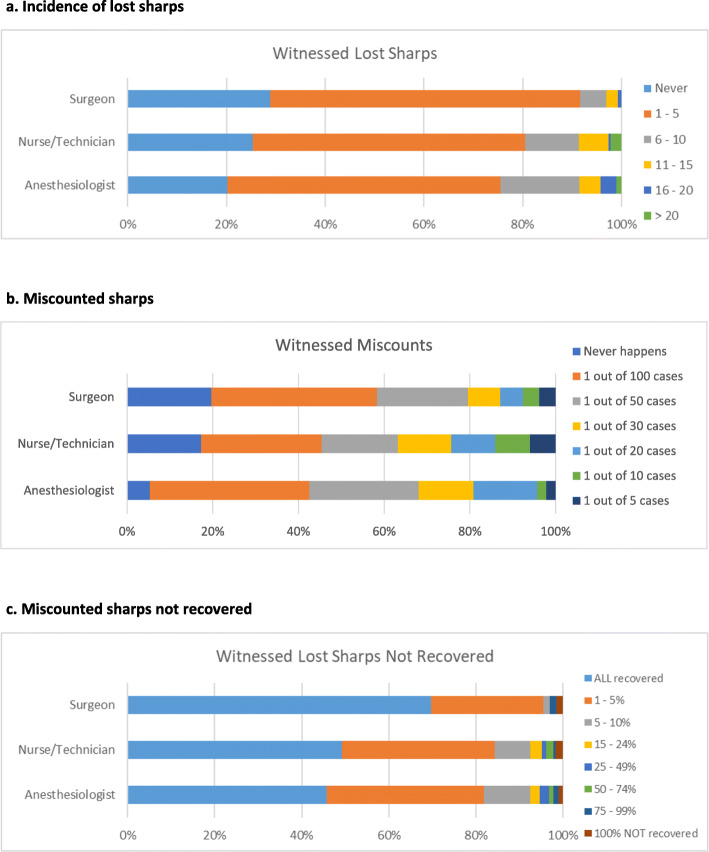
**a** Incidence of lost sharps. **b** Miscounted sharps. **c** Miscounted sharps not recovered

When evaluating the incidence of lost sharps over the last year (question #4), there was an association between the respondent’s role and the number of reported lost sharps. Overall, most of each respondent group reported 1–5 lost sharp events over the last year (91.7% of surgeons, 75.5% of anesthesiologists and 80.5% of nurses/technologists) (Fig. 2a).

Questions 5 revealed the incidence of miscounted sharps in the last year (Fig. 2b). Significant discordance was found between the surgeon and anesthesiologist group regarding how often they believe miscounts “never” happen. Here, roughly 20% of surgeons believed they never had a miscounted sharp over the last year, where only 5.3% of anesthesiologist reported the same (*p* = 0.002).

Regarding the number of miscounted sharps not recovered prior to the completion of the surgery (question #6, Fig. 2c), 30.3% of surgeons, 54.3% of anesthesiologists, and 50.8% of nurses/technologists reported that not all miscounted sharps were recovered prior to completion of the surgery. Statistically significant differences were found between the anesthesiologist vs surgeon (*p* < 0.001), and nurse/technologist vs surgeon (*p* < 0.001).

No significant difference (*p* = 0.519) was found between the three respondent groups when evaluating the number of NMS per 1000 surgeries and reported as 3.77 (anesthesiologists), 5.06 (surgeons) and 4.28 (nurses/technologists). A significant difference was found between the three groups regarding the number of lost sharps not recovered per 10,000 surgeries with anesthesiologist, surgeon and nurse/technologist groups estimating 2.37, 2.56 and 2.94 respectively (*p* = 0.001) (Table 3).

**Table 3:**
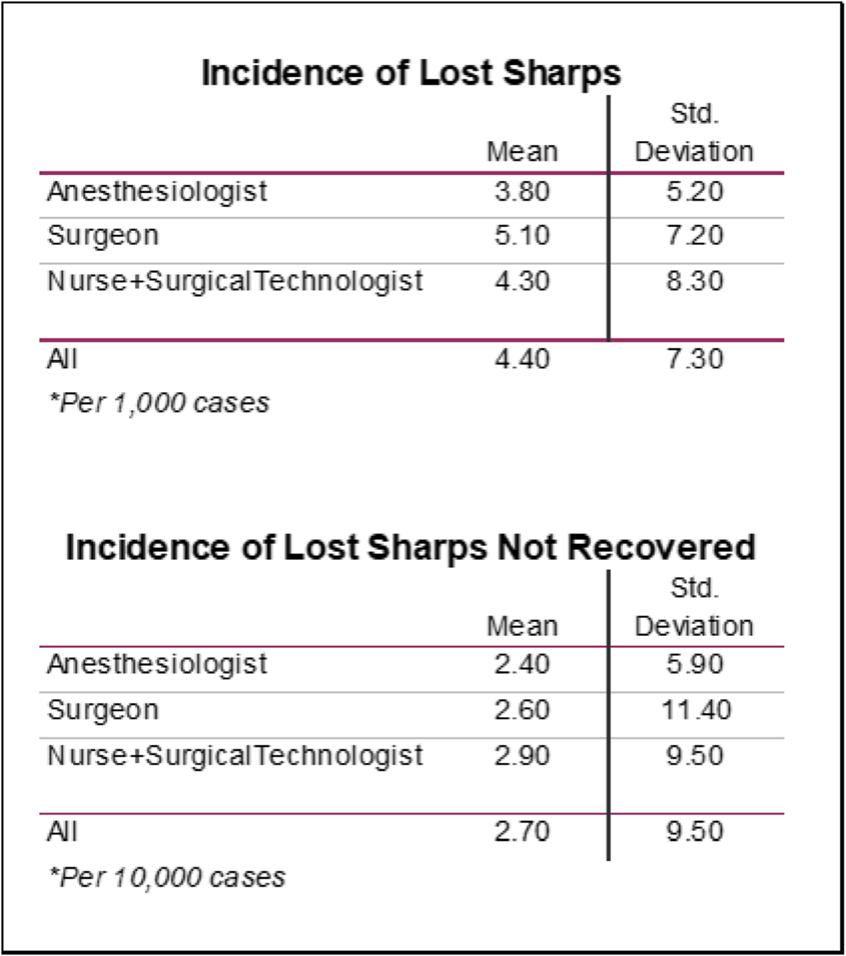
Incidence of lost sharps and lost sharps not recovered

### ***X***-ray use and effectiveness

**Fig. 3:**
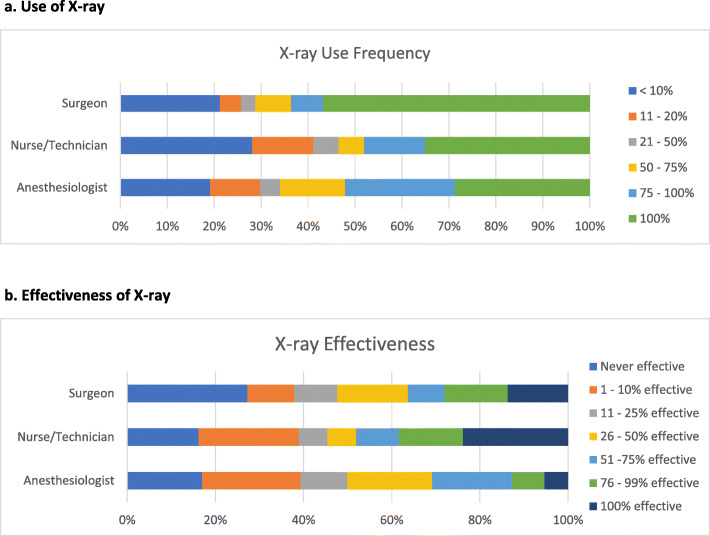
**a** Use of X-ray. **b** Effectiveness of X-ray

Discordance was found in the perception of how frequently portable x-ray was used to aid in the recovery of lost sharps (question #8, Fig. 3a). Here, more than half (56.8%) of surgeons report using x-ray 100% of the time when managing a lost sharp whereas anesthesiologists and nurses/technologists believe it is closer to 1/3 of the time. Agreement was found between all three groups when describing the effectiveness of x-ray and believed to be between 26 and 50% effective in identifying a lost sharp. Additionally, roughly 38% of each group reported that it is never effective or effective only 1–10% of the time (Fig. 3b).

### ***T***ime spent managing surgical sharps

Time added to the OR due to x-ray was found to be similar between the three respondent groups at 31-40 min. Additionally, 21–30 min was reported as the median rank for time spent searching for a lost sharp.

### Recall bias

The binomial logistic regression performed was statistically significant, with *p* values of 0.002, 0.003, < 0.001 and < 0.001 for terms 1–4 respectively. Additionally, surgeons were 2.95, 2.49 and 2.67 times more likely to respond in the affirmative to statements 1, 3 and 4 respectively compared to the collective nurse/technologist/anesthesiologist group. In contrast, the surgeon group was 0.475 times less likely to report miscounting of sharps in > 2% of surgeries within the last year when compared to the nurse/technologist/anesthesiologist group (term #2).

## Discussion

To our knowledge, this is the first anonymous survey conducted to estimate the degree of NMS and RSS events. Several key ideas were illustrated by the survey responses, including incidence within the last year, incidence per number of surgical cases, surgical team agreement, and surgical team discordance.

While most literature estimates a retained foreign body event to occur once out of every 1000 to 18,000 surgeries (with poor delineation between a sharps vs other foreign bodies) [9–11], our data suggest a higher incidence of 2.7 events per 10,000 surgeries (roughly 1 event per every 3800 surgeries). This further supports the concern for the underreporting of RSS. Additionally, respondents experience 2.7–4.3 lost sharp events each year (4.3 - anesthesiologist, 2.7 - surgeons, 3.9 - nurses/technologists) with an average incidence of 4.4 lost sharp events per every 1000 surgeries. The underreported nature of NMS and RSS may be attributed to the challenges associated with reporting and concern for negative repercussions on the provider or surgical team.

In addition to underreporting, the survey demonstrates significant discordance between surgeons’ perceptions and those of the other OR team members suggesting that recall bias may play a significant role in better defining the scope of the sharps problem. Recall bias would be expected as the surgeon plays the primary role in the operating room and bears the bulk of responsibility and potential negative repercussions. Therefore, an odds ratio was obtained comparing surgeons to the remainder of the surgical team (anesthesia, nursing, and technologists) to determine if there was a difference in perception of the number of events present in those conducting the surgery vs those participating in the surgery. Notably, surgeons were found to be 2.95 times more likely to report zero lost events in the last year. Additionally, surgeons were found to be 2.5 times more likely to report 100% of all lost sharps recovered before the completion of the surgery. Consistent with the previously mentioned odds ratios, the surgeon group was half as likely to report miscounting of sharps in > 2% of surgeries. This may be because the surgeons are not directly involved in the instrument counting process thereby increasing their recall bias. Together these odds ratios support the conclusion that surgeons are less likely to perceive that a sharp has been lost and more likely to perceive that all lost sharps have been recovered, suggesting a larger recall bias as compared to the rest of the operative team.

With each lost/retained sharp event there are significant implications on the health system, which encompasses the patient, provider, and hospital. Patient implications include additional exposure to radiation as well as the prolonged anesthesia time and increased risk for iatrogenic damage during search and recovery. Prolonged operative time has been significantly associated with increased risk for infection and additional post-surgical complications as well as prolonged hospital length of stay [12–14]. As such, investigating risks to the patient was a focal point of this study.

Agreement was found between all three groups of respondents when evaluating the amount of time added to the OR as a result of manually searching for a lost sharp (21-30 min) as well as by the time added to the OR when obtaining an x-ray (31-40 min). This concordance suggests that each lost sharp event may result in up to 70 min of added OR time.

The survey results also highlighted the need for improved technologies in identifying RSS, as 69.1% of anesthesiologists, 63.6% of surgeons, and 51.9% of nurses/technologists said that an x-ray is 1–50% effective, with approximately 38% of each group reporting that it is never effective or effective only 1–10% of the time. Prior studies have evaluated the effectiveness of x-ray in identifying lost sharps, and more specifically the effectiveness at identifying needles. They note needle size should act as a key determining factor when deciding on whether an x-ray should be obtained to aid in needle recovery, quoting poor effectiveness in needles < 17 mm [15]. When evaluating the prolonged operative time associated with the use of x-ray (31-40 min) as well as the cost of the x-ray and radiation exposure to the patient, x-ray appears to be a costly and ineffective method of identifying RSS.

While patient implications are paramount, we would be remiss if we failed to mention the psychological and economic impact of a lost/retained sharps on the provider and the health system. The provider is faced with personal, professional, and financial repercussions in the event of a RSS, especially in the setting of patient injury. Given that a retained surgical instrument is classified as a never event, there is no defense from a litigation standpoint and the provider must shoulder the blame and consequences of the event [9]. Being responsible for harm coming to a patient is psychologically damaging and the results of the far-reaching effects on the surgeon have been termed “second victim syndrome” [16]. Anxiety, guilt, sadness, shame, and embarrassment have all been reported by physicians after experiencing an intraoperative adverse event, and the added stress of such an event increases the chances of further mistakes [16]. Therefore, it is unsurprising that the surgeon group demonstrated a greater likelihood of recall bias related to frequency and recovery of RSS when compared to the anesthesia and nurse/technologist groups, as this bias is self-protective against a challenging situation that can result in damage to their professional reputation, financial loss through litigation, and emotional distress.

The hospital is also subject to negative consequences in the setting of a RSS as costs associated with resulting complications are not reimbursable, with the average cost per patient ranging from 70,000–200,000 [7, 17]. In the case of a settlement, costs to the hospital can range from 2 to 5 million dollars even with a positive patient outcome [7].

A team-based approach is paramount to reducing the barriers to reporting these events and is appropriate given that over 90% of retained items are due to a team/system-based error [18, 19]. Here we see a significant degree of discordance between the different members of the operative team providing initial evidence that OR staff may perceive the frequency of lost, miscounted and retained surgical sharps differently. This hints at a lack of communication regarding these sentinel events creating opportunity for undue patient harm. By creating an environment of transparency and a standardized reporting system, helpful discussions of ways to prevent retained surgical sharps in the future could be had between all members of the team and reduce the risks to the patient, physician, and hospital.

The estimated value of incidence would be a limitation of present study. The incidence value was derived by taking the median value of each response term which was originally in a range, and this may result in a relatively inaccurate value to present the mean scores and interpretation of the differences between groups. While more granular detail regarding respondent demographics or the type of sharp that was lost/retained/miscounted could have been collected, and therefore act as a study limitation, we aimed to keep the survey short to increase the number of responses. Additionally, while team discordance is suggested by this study, respondents were not necessarily from the same surgical team and therefore the effectiveness of any one surgical team’s communication cannot be derived from this study.

## Conclusions

NMS and RSS are more prevalent than what is reported in current literature. Surgeons perceive a higher rate of success in retrieving the RSS when compared to anesthesiologists and nurses/OR technologists. We recognize several challenges surrounding “near-miss” and never events as contributing factors to their underreported nature and the higher degree of surgeon recall bias associated with these events. Additionally, we highlight that current methods for prevention of NMS and RSS are costly in time and resources without improvement in patient safety. As NMS and RSS have significant health system implications, a strong understanding of these implications is important as we strive to improve patient safety.

## Data Availability

The data used in this study can be made available upon written request to Samuel.Weprin@vcuhealth.org.

## Abbreviations

RSS: Retained surgical sharp
NMS: Near-miss sharp
PSC: Patient Safety Culture
OR: Operating Room
JC: Joint Commission
